# Cognitive impairment 2 years after mild to severe SARS-CoV-2 infection in a population-based study with matched-comparison groups

**DOI:** 10.1038/s41598-025-96608-0

**Published:** 2025-07-08

**Authors:** Natália Araújo, Isa Silva, Patrícia Campos, Adriana Costa, Catarina Lopes, Mariana Seco, Ana Rute Costa, Maria Margarida Calejo, Maria Joana Pais, Susana Pereira, Samantha Morais, João Firmino Machado, Luís Ruano, Nuno Lunet, Vítor Tedim Cruz

**Affiliations:** 1 EPIUnit ITR, Instituto de Saúde Pública da Universidade do Porto, Porto, Portugal; 2 Departamento de Ciências da Saúde Pública e Forenses, e Educação Médica, Faculdade de Medicina da Universidade do Porto, Porto, Portugal; 3 Unidade de Neuropsicologia, Unidade Local de Saúde de São João, E.P.E, Porto, Portugal; 4 Serviço de Neurologia, Unidade Local de Saúde de Matosinhos, E.P.E, Senhora da Hora, Portugal; 5 Serviço de Neurologia, Instituto Português de Oncologia do Porto, Dr. Francisco Gentil, E.P.E, Porto, Portugal; 6 Departamento de Ciências Médicas, Universidade de Aveiro, Aveiro, Portugal; 7 Serviço de Neurologia, Unidade Local de Saúde de Entre Douro e Vouga, E.P.E, Santa Maria da Feira, Portugal

**Keywords:** Cognitive impairment, SARS-CoV-2 infection, COVID-19, Neuropsychological assessment, Neurology, Risk factors

## Abstract

COVID-19 may have long lasting cognitive consequences. Studies with a follow-up longer than 1 year after infection are lacking. This study presents the prevalence of cognitive impairment 2 years after SARS-CoV-2 infection in survivors of the first year of the pandemic and comparison groups matched 1:1 for sex, age, and level of care. Users of the Local Health Unit of Matosinhos (comprising almost all citizens of the municipality) were retrospectively selected according to hospitalization and SARS-CoV-2 infection between March 2020 and February 2021: group #1, hospitalized for COVID-19 (n = 101); group #2, hospitalized, uninfected (n = 87); group #3, non-hospitalized, infected (n = 252); group #4, non-hospitalized, uninfected (n = 258). Between July 2022 and October 2023, all participants completed the Montreal Cognitive Assessment. Those with a score below 1.5 SD of age- and education-specific norms (n = 279) were invited for a comprehensive neuropsychological assessment to identify cognitive impairment. The prevalence of cognitive impairment was higher in group #1 than #2 (19.1% vs. 6.8%; adjusted OR 5.41, 95% CI 1.54, 19.03) and in group #3 than #4 (10.7% vs. 3.2%; adjusted OR 3.27, 95% CI 1.23, 8.67). These results suggest that specific care to timely diagnose and treat cognitive impairment is needed for COVID-19 survivors of the first year of the pandemic.

**Supplementary Information:**

The online version contains supplementary material available at 10.1038/s41598-025-96608-0.

## Introduction

COVID-19 no longer constitutes a public health emergency of international concern^1^. However, a significant proportion of the population was exposed to COVID-19 in the first year of the pandemic. In this period, individual and group immunities to the disease were low and SARS-CoV-2 infection caused severe disease more frequently^2^.

Previous studies have reported higher risks of neurodegenerative diseases following the acute phase of SARS-CoV-2 infection compared to SARS-CoV-2-uninfected individuals^3^. These risks were even higher in the most severe cases of COVID-19 and in unvaccinated individuals^4^. Conversely, deficits in cognitive tests were reported to be higher in individuals with COVID-19 post-acute sequelae persistent for more than 12 weeks than in SARS-CoV-2-uninfected individuals. Moreover, larger deficits were noticed in survivors of COVID-19 who had been hospitalized and whose infection occurred in periods when original and alpha variants were dominant^5^. However, methodological issues have limited our understanding of the effect of COVID-19 on cognitive function, specifically after a period of more than 1 year following infection. Indeed, the validity of previous results is limited due to the possibility of misclassification of neurological outcomes in studies based on electronic health records and weak control of confounding, and the lack of comparison groups and robust assessment of cognitive performance. Additionally, there has been a focus on patients hospitalized due to COVID-19 or experiencing post-COVID-19 conditions, with few results on less severe COVID-19. Finally, results have not consistently pointed to an effect of COVID-19 on cognitive performance^3,4,6^.

The NeurodegCoV-19 prospective study aims to compare the cognitive trajectories of individuals infected with SARS-CoV-2 (hospitalized and non-hospitalized) and comparison groups from nearly 2 years after SARS-CoV-2 infection with a 1 year follow-up^7^. The present study presents the prevalence of cognitive impairment at the baseline evaluation of the cohort and explores its association with SARS-CoV-2 infection. It allows us to understand if survivors of COVID-19 from the first year of the pandemic are more likely to present cognitive impairment 2 years after SARS-CoV-2 infection than the general population.

## Methods

### Setting and participants

This study uses information from the baseline evaluation of participants of the NeurodegCoV-19 cohort study described previously^7^.

In brief, the study population was the county of Matosinhos, North Portugal, the most affected region by the first wave of the COVID-19 pandemic. Nearly all its citizens are registered at the *Unidade Local de Saúde de Matosinhos* (ULSM). ULSM comprises a 380-bed hospital, several primary care units, and palliative care units, serving as the sole provider of primary and hospital care in Matosinhos, within the Portuguese National Health System. Based on hospital admission episodes and SARS-CoV-2 infection status from March 2020 to February 2021, we identified four study groups: group #1—individuals hospitalized due to COVID-19; group #2—hospitalized individuals without a positive test for SARS-CoV-2 infection; group #3—individuals followed in the community after a positive test for SARS-CoV-2 infection; and group #4—non-hospitalized individuals without a positive test for SARS-CoV-2 infection. Groups #1 and #2 were matched for age (10-year categories), sex, and level of care (hospital admission to intensive care units, intermediate care units, or general wards). Groups #3 and #4 were matched for age (10-year categories) and sex. Inclusion and exclusion criteria are described in Supplementary Table S1 and the selection process of participants is presented in Supplementary Figs. S1–S5.

Our comparison groups consist of individuals who have never been infected with SARS-CoV-2 and those who became infected at least 14 days after completing their primary COVID-19 immunization (Supplementary Table S2).

### Procedures

In-person evaluations of participants included a structured interview to collect data on sociodemographic characteristics, namely sex, age, education, marital status, and employment, lifestyles (smoking behavior and alcohol consumption further categorized based on recommended thresholds—20 g/day for males younger than 65 years and 10 g/day otherwise)^8,9^, weight and height, symptoms during and after SARS-CoV-2 infection, and current symptomatology, comorbidities, medication, COVID-19 vaccination status, and dates of SARS-CoV-2 infections after February 2021. The Portuguese-validated versions of the Hospital Anxiety and Depression Scale (HADS)^10,11^ and Sleep Quality Index (PSQI)^12,13^ were used for the assessment of anxiety and depression symptoms, and sleep quality, respectively. Scores above ten for the HADS-anxiety (HADS-A) and HADS-depression (HADS-D) subscales^10,11^, and above five for the PSQI^12,13^ were used to define clinically significant anxiety and depression symptoms, and poor sleep quality, respectively.

Cognitive assessment was conducted in two steps: (1) all participants completed the Portuguese-validated version of the Montreal Cognitive Assessment (MoCA)^14,15^; (2) those with a score at least 1.5 standard deviations (SD) below age- and education-specific means from normative data^16^ were invited to perform a neuropsychological assessment conducted by one of four psychologists with the same specific training in neuropsychology. Verbal memory, visual memory, executive functions, language, and information processing speed and attention were evaluated using the tests described in Supplementary Table S3. Cognitive impairment in a given domain was determined based on criteria that consider the number of scores used to assess each cognitive domain, reducing the risk of overestimating deficits due to chance^17^. A participant was considered to have cognitive impairment when there was impairment in at least one cognitive domain. From this two-step assessment, participants were classified as normal–normal performance in the MoCA or in the neuropsychological assessment—or with cognitive impairment—low performance in the MoCA and cognitive impairment identified in the neuropsychological assessment. Participants with an abnormal performance in the MoCA but who did not perform the neuropsychological assessment were excluded from the primary analysis and included in a secondary analysis.

Additionally, the proportion of participants with a MoCA score below 2 SD, and below 22 and 17, the cutoffs to identify mild cognitive impairment and dementia, respectively, in the Portuguese population^18–20^, were compared between groups #1 versus #2, and groups #3 versus #4.

### Statistical analysis

Summary descriptors, namely mean and SD for continuous variables and absolute and relative frequencies for categorical variables, were used to describe the four study groups. A T-test for independent samples and Pearson’s Chi-square test were used to compare groups #1 versus #2, and groups #3 versus #4 (Table 1).

**Table 1:** Sociodemographic characteristics and lifestyles of participants according to hospitalization and SARS-CoV-2 infection status between March 2020 and February 2021.

	Hospitalized		*P* value	Non-hospitalized		*P* value
	For COVID-19	Uninfected		Infected	Uninfected	
	Group #1	Group #2		Group #3	Group #4	
	n (%)	n (%)		n (%)	n (%)	
Sociodemographic characteristics						
Age (years)			0.446			0.998
< 25	0 (0.0)	0 (0.0)		7 (2.8)	8 (3.1)	
25–34	1 (1.0)	0 (0.0)		20 (7.9)	22 (8.5)	
35–44	2 (2.0)	1 (1.1)		42 (16.7)	41 (15.9)	
45–54	11 (10.9)	4 (4.6)		66 (26.2)	70 (27.1)	
55–64	30 (29.7)	25 (28.7)		61 (24.2)	57 (22.1)	
65–74	44 (43.6)	40 (46.0)		43 (17.1)	46 (17.8)	
> 74	13 (12.9)	17 (19.5)		13 (5.2)	14 (5.4)	
Education (years)			**0.021**			0.452
≤ 4	47 (46.5)	45 (51.7)		51 (20.2)	42 (16.3)	
5–9	17 (16.8)	25 (28.7)		60 (23.8)	54 (20.9)	
10–12	23 (22.8)	7 (8.0)		52 (20.6)	62 (24.0)	
> 12	14 (13.9)	10 (11.5)		89 (35.3)	100 (38.8)	
Sex			0.777			0.995
Females	34 (33.7)	31 (35.6)		171 (67.9)	175 (67.8)	
Males	67 (66.3)	56 (64.4)		81 (32.1)	83 (32.2)	
Marital status			**0.041**			0.391
Single	5 (5.0)	2 (2.3)		32 (12.7)	42 (16.3)	
Married/living with a partner	76 (75.2)	54 (62.1)		179 (71.0)	169 (65.8)	
Widowed/divorced/separated	20 (19.8)	31 (35.6)		41 (16.3)	46 (17.9)	
Employment			**0.019**			0.799
Employed	38 (37.6)	19 (21.8)		170 (67.7)	172 (66.7)	
Unemployed/retired/on sick leave	63 (62.4)	68 (78.2)		81 (32.3)	86 (33.3)	
Lifestyles						
Alcohol consumption			0.917			**0.035**
≤ 20 g/day^a^	83 (82.2)	72 (82.8)		231 (91.7)	248 (96.1)	
> 20 g/day	18 (17.8)	15 (17.2)		21 (8.3)	10 (3.9)	
Smoking			**0.006**			**0.024**
Never smoker	54 (53.5)	36 (41.4)		140 (55.6)	135 (52.3)	
Ex-smoker	44 (43.6)	37 (42.5)		78 (31.0)	65 (25.2)	
Current smoker	3 (3.0)	14 (16.1)		34 (13.5)	58 (22.5)	

Significant values are in [bold].

^a^For males aged 65 or older and all females, this value is reduced to 10 g/day, according to recommendations of the Portuguese Health Authority, *Direção Geral de Saúde.*

Potential confounders (Supplementary Table S4) of the association between study groups and cognitive impairment were identified based on the existing literature, namely risk factors of SARS-CoV-2 infection^21,22^, and using Directed Acyclic Diagrams (available at: https://dagitty.net/mdGEJQnCH).

Multivariate logistic regression models were used to estimate odds ratios (OR) and respective 95% confidence intervals (95% CI) of the associations between participants’ characteristics, namely study group, and cognitive impairment, including potential confounders as covariates, as specified in each model in the footnotes of Fig. 2. The link, and Hosmer and Lemeshow’s goodness-of-fit tests were used to confirm that logistic regression models were correctly specified. Multicollinearity problems were also checked considering a Variance Inflation Factor above 2.5.

Statistical analyses were performed using Stata, version 15.1 (StataCorp, College Station, TX, USA), and a significance level of 0.05 was considered.

As a secondary analysis, predicted probabilities of cognitive impairment among participants with abnormal MoCA scores who did not perform the neuropsychological assessment were computed based on the multivariate logistic regression model with cognitive impairment as the dependent variable, and education, comorbidities, HADS-A, HADS-D, and PSQI scores, employment, and study group, as independent variables. This model presented an acceptable accuracy to detect cognitive impairment (area under the receiver operating characteristic curve of 0.793). The predicted probabilities of cognitive impairment were compared between groups #1 versus #2 and groups #3 versus #4 with the Kolmogorov–Smirnov test.

### Ethics

This study follows the ethics principles of the World Medical Association Declaration of Helsinki and the General Data Protection Regulation and was approved by the Ethics Committee from the Local Health Unit of Matosinhos (ref. 117/CES/JAS, September 17, 2021) and its committee for data protection (ref. 030/CLPSI/2021, May 03, 2022). All participants signed the written informed consent.

## Results

### Description of the groups

A total of 698 participants were included in the present analysis, grouped according to hospitalization and positivity for SARS-CoV-2 infection between March 2020 and February 2021. From all survivors of COVID-19 who had been hospitalized due to COVID-19, we included all 101 eligible who accepted to participate (group #1); among hospitalized individuals without a positive test for SARS-CoV-2 infection, we were able to match 87 with group #1, for sex and age, matching for hospital level of care was not fully complete due to insufficient cases being admitted to the intensive care unit (group #2); a total of 252 individuals who had been followed in the community after a positive test for SARS-CoV-2 infection (group #3), and 258 who had not been hospitalized nor had a positive test for SARS-CoV-2 infection (group #4) were included. The baseline evaluation was performed between July 2022 and October 2023, a median (25th percentile; 75th percentile) 705 days (671; 818) and 670.5 days (652; 701) after the first positive test for SARS-CoV-2 infection in groups #1 and #3, respectively. In group #2, a median time (25th percentile; 75th percentile) of 1006 days (941; 1117) had passed since hospital admission when the baseline evaluation was performed. No index date was considered for group #4.

In groups #2 and #4, 39.5% and 63.9% of participants, respectively, reported at least one positive test for SARS-CoV-2 infection after complete primary COVID-19 vaccination. The first positive test was dated after January 2022 for most of these participants (Supplementary Table S2).

Male participants were similarly frequent in groups #1 and #2 (66.3% and 64.4%), and groups #3 and #4 (32.1% and 32.2%. Mean age (SD) was 65.2 (10.0) and 67.9 (8.7) years in groups #1 and #2, and 52.8 (14.1) and 52.9 (14.1) years in groups #3 and #4, respectively. Educational level was similar between groups #3 and #4 (55.9% and 62.8% had at least 10 years of schooling, *P* = 0.452), but there were more participants with less than 10 years of schooling in group #2 than #1 (80.5% and 63.3%, respectively; *P* = 0.021). Group #2 had more widowed, divorced or separated (35.6% vs. 19.8%, *P* = 0.041), and unemployed, retired or on sick leave (78.2% vs. 62.4%, *P* = 0.019) participants than group #1, whereas group #3 was similar to group #4 regarding marital status (widowed, divorce or separated: 16.3% vs. 17.9%, *P* = 0.391) and employment (Unemployed/Retired/On sick leave: 32.3% vs. 33.3%, *P* = 0.799). Consumption of alcohol above age- and sex-recommended thresholds was observed more frequently in group #3 than #4 (8.3% vs. 3.9%, *P* = 0.035) and similar between groups #1 and #2 (17.8% vs 17.2%, *P* = 0.917). In contrast, current smokers were more frequent in group #2 than #1 (16.1% vs. 3.0%, *P* = 0.006) and in group #4 than #3 (22.5% vs. 13.5%, *P* = 0.024) (Table 1).

Cancer and chronic obstructive pulmonary disease were more prevalent in group #2 than #1 (26.4% vs. 14.9%, *P* = 0.049, and 4.6% vs. 0.0%, *P* = 0.029, respectively), and liver disease was more frequent in group #3 than #4 (8.3% vs. 3.9%, *P* = 0.035). The consumption of more than two medications per day was higher in group #2 than #1 (81.6% vs. 66.3%, *P* = 0.018) and similar between groups #3 and #4 (40.5% vs. 37.60%, *P* = 0.505). The consumption of drugs used in the Nervous System (Anatomical Therapeutic Chemical Classification N) was similar between the groups of hospitalized participants (41.6% and 42.5%, *P* = 0.896) and in groups #3 and #4 (28.2% and 29.1%, *P* = 0.711) (Table 2).

**Table 2:** Comorbidities and medicines consumption in participants grouped according to hospitalization and SARS-CoV-2 infection status between March 2020 and February 2021.

	Hospitalized		*P* value	Non-hospitalized		*P* value
	For COVID-19	Uninfected		Infected	Uninfected	
	Group #1	Group #2		Group #3	Group #4	
	n (%)	n (%)		n (%)	n (%)	
Comorbidities						
Hypertension	67 (66.3)	55 (63.2)	0.655	94 (37.3)	78 (30.2)	0.091
Hypercholesterolemia	62 (61.4)	57 (65.5)	0.558	83 (32.9)	89 (34.5)	0.710
Obesity	47 (46.5)	31 (35.6)	0.130	41 (16.3)	39 (15.1)	0.706
Diabetes type II	30 (28.7)	23 (26.4)	0.533	36 (14.3)	28 (10.9)	0.235
Glaucoma	2 (2.0)	1 (1.1)	0.650	3 (1.2)	6 (2.3)	0.333
Retinopathy	1 (1.0)	1 (1.1)	0.915	6 (2.4)	7 (2.7)	0.823
Angina pectoris	2 (2.0)	0 (0.0)	0.187	2 (0.8)	3 (1.2)	0.676
Acute myocardial infarction	2 (2.0)	5 (5.7)	0.174	2 (0.8)	4 (1.6)	0.431
Heart failure	5 (5.0)	7 (8.0)	0.387	3 (1.2)	4 (1.6)	0.731
Arrhythmia	3 (3.0)	0 (0.0)	0.105	28 (11.2)	21 (8.1)	0.249
Another heart disease	23 (22.8)	22 (25.3)	0.687	17 (6.7)	17 (6.6)	0.943
Peripheral arterial disease	1 (1.0)	3 (3.4)	0.244	2 (0.8)	5 (1.9)	0.269
Stroke	1 (1.0)	4 (4.6)	0.125	3 (1.2)	2 (0.8)	0.631
Transient ischemic attack	0 (0.0)	1 (1.1)	0.280	7 (2.8)	3 (1.2)	0.186
Brain aneurism	0 (0.0)	0 (0.0)	n/a	2 (0.8)	0 (0.0)	0.151
Epilepsy	0 (0.0)	0 (0.0)	n/a	11 (4.4)	4 (1.6)	0.059
Another neurological disease	4 (4.0)	1 (1.1)	0.232	2 (0.8)	2 (0.8)	0.978
Anxiety	10 (9.9)	5 (5.7)	0.295	66 (26.3)	58 (22.5)	0.316
Depression	19 (18.8)	12 (13.8)	0.411	69 (27.5)	65 (25.4)	0.592
Another psychiatric problem	0 (0.0)	1 (1.1)	0.280	7 (2.8)	2 (0.8)	0.088
Cancer	15 (14.9)	23 (26.4)	**0.049**	20 (8.0)	29 (11.2)	0.216
Asthma	7 (6.9)	4 (4.6)	0.497	27 (10.8)	25 (9.7)	0.702
Rhinitis	4 (4.0)	6 (6.9)	0.371	55 (21.9)	46 (17.8)	0.248
Pulmonary emphysema	1 (1.0)	0 (0.0)	0.352	1 (0.4)	4 (1.6)	0.188
Chronic bronchitis	0 (0.0)	0 (0.0)	n/a	7 (2.8)	6 (2.3)	0.740
Chronic obstructive pulmonary disease	0 (0.0)	4 (4.6)	**0.029**	2 (0.8)	2 (0.8)	0.978
Obstructive sleep apnea	19 (18.8)	12 (13.8)	0.355	12 (4.8)	5 (1.9)	0.074
Hypoacusia	3 (3.0)	1 (1.1)	0.388	10 (4.0)	10 (3.9)	0.950
Hypothyroidism	3 (3.0)	3 (3.4)	0.853	9 (3.6)	6 (2.3)	0.401
Hyperthyroidism	0 (0.0)	3 (3.4)	0.060	15 (6.0)	10 (3.9)	0.277
Osteoporosis	5 (5.0)	4 (4.6)	0.910	15 (6.0)	16 (6.2)	0.915
Osteoarthritis	16 (15.8)	18 (20.7)	0.389	51 (20.3)	39 (15.1)	0.124
Rheumatoid arthritis	1 (1.0)	0 (0.0)	0.352	4 (1.6)	6 (2.3)	0.548
Lupus	1 (1.0)	1 (1.1)	0.915	0 (0.0)	1 (0.4)	0.323
Hernia	12 (11.9)	11 (12.6)	0.874	50 (20.0)	41 (15.9)	0.227
Urinary incontinence	6 (5.9)	12 (13.8)	0.068	30 (12.0)	34 (13.2)	0.677
Kidney disease	15 (14.9)	20 (23.0)	0.153	27 (10.8)	17 (6.6)	0.094
Gastric disease	25 (24.8)	32 (36.8)	0.074	45 (17.9)	49 (19.0)	0.741
Liver disease	9 (8.9)	9 (10.3)	0.739	21 (8.3)	10 (3.9)	**0.035**
Allergies	2 (2.0)	1 (1.1)	0.650	52 (20.8)	40 (15.5)	0.121
Another disease	49 (48.5)	47 (54.0)	0.451	84 (33.9)	91 (35.3)	0.741
Medicines consumption						
Number of medicines^a^			0.075			0.711
None	9 (8.9)	7 (8.0)		73 (29.0)	87 (33.7)	
1–2/day	25 (24.8)	9 (10.3)		77 (30.6)	74 (28.7)	
3–4/day	28 (27.7)	30 (34.5)		66 (26.2)	64 (24.8)	
≥ 5/day	39 (38.6)	41 (47.1)		36 (14.3)	33 (12.8)	
Median (25th, 75th percentiles)	4 (2,8)	5 (3,7)	0.211	2 (0,4)	1 (0,4)	0.936
Consumption of at least one medicine of the ATC class						
Drugs used in diabetes^b^	30 (29.7)	29 (33.3)	0.593	37 (14.7)	29 (11.2)	0.247
Drugs used in hypertension^b,c^	65 (64.4)	59 (67.8)	0.618	75 (29.8)	77 (29.8)	0.984
Lipid-modifying agents^b^	45 (44.6)	49 (56.3)	0.108	64 (25.4)	69 (26.7)	0.729
Drugs used in the nervous system^b^	42 (41.6)	37 (42.5)	0.896	71 (28.2)	75 (29.1)	0.823
Psychoanaleptics^b^	23 (22.8)	18 (20.7)	0.730	46 (18.3)	40 (15.5)	0.407
Antidepressants^b^	22 (21.8)	18 (20.7)	0.855	45 (17.9)	40 (15.5)	0.476
Psycholeptics^b^	21 (20.8)	18 (20.7)	0.986	30 (11.9)	37 (14.3)	0.415
Benzodiazepines^b^	18 (17.8)	15 (17.2)	0.917	24 (9.5)	30 (11.6)	0.440

Significant values are in [bold].

^a^Except contraceptives; medicines that are a combination of drugs were counted as one.

^b^Medicines containing one or a combination of drugs were classified according to the anatomical therapeutic chemical (ATC) classification.

^c^This category of medicines includes the ATC codes for antihypertensives (C02), diuretics (C03), beta blocking agents (C07), calcium channel blockers (C08), agents acting on the renin-angiotensin system (C09).

Length of hospitalization in days was similar between groups #1 and #2. There were more participants admitted to the intensive care unit in group #1 than #2 (24.8% vs. 11.5%, *P* = 0.023) despite all efforts to achieve matching (Table 3).

**Table 3:** Characteristics of the hospital admission, according to the SARS-CoV-2 infection status.

	Hospitalized for COVID-19	No positive test for SARS-CoV-2 infection	*P* value
	Group #1	Group #2	
	n (%)	n (%)	
Length of stay (days)			0.326
< 1	3 (3.0)	0 (0.0)	
1	1 (1.0)	2 (2.3)	
2–7	20 (19.8)	21 (24.1)	
8–15	51 (50.50)	47 (54.0)	
16–30	17 (16.8)	14 (16.1)	
> 30	9 (8.9)	3 (3.5)	
Level of care			**0.023**
Intensive care unit	25 (24.8)	10 (11.5)	
Intermediate care unit	19 (18.8)	12 (13.8)	
General ward	57 (56.4)	65 (74.7)	

Significant values are in [bold].

### MoCA classification of participants

Mean values [SD] of MoCA scores were similar between groups #1 and #2 (20.6 [4.8] and 20.5 [4.5]; *P* = 0.886), but lower in group #3 than #4 (23.4 [4.4] and 24.1 [3.6], *P* = 0.036).

**Fig. 1:**
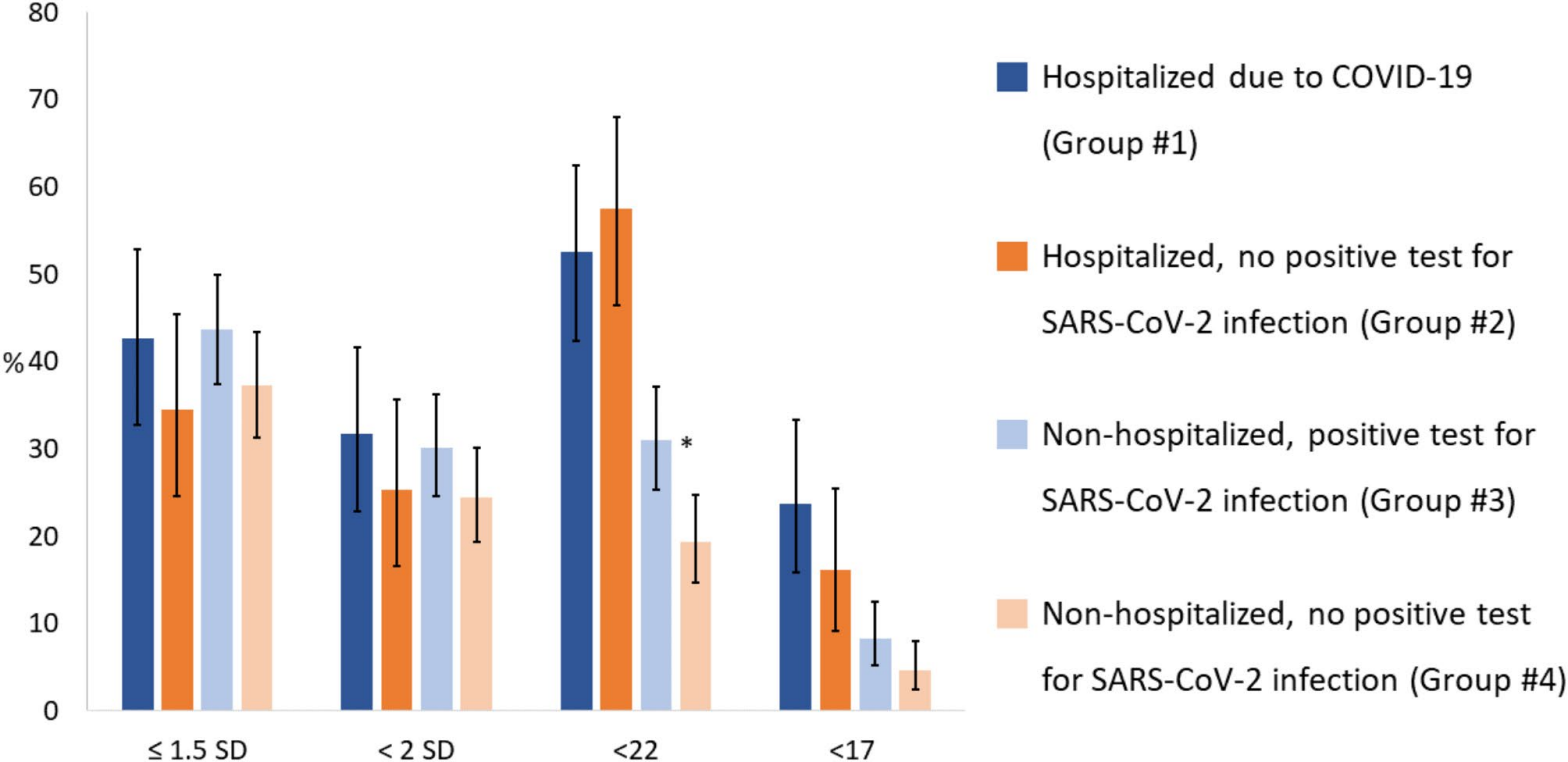
Proportion of participants in each group (based on hospitalization and SARS-CoV-2 infection status between March 2020 and February 2021) with a Montreal Cognitive Assessment (MoCA) score suggesting probable cognitive dysfunction. Probable cognitive dysfunction was identified as a MoCA score below what is expected in the general population, using the cutoffs for moderate or severe dysfunction, whenever the score was at least 1.5 standard deviations (SD) or 2 SD, respectively, below the mean of age- and education-specific norms (published normative data in the Portuguese population^16^); or a MoCA score below specific cutoffs for screening mild cognitive impairment (cutoff 22) or dementia (cutoff 17) obtained from studies in the Portuguese population^18–20^. Vertical error bars correspond to 95% confidence intervals. *P = 0.003, Chi-square test comparing groups #3 and #4.

The proportions of participants with a MoCA score below 1.5 SD of the mean based on the age- and education-specific norms were 42.6%, 34.5%, 43.7%, and 37.2% in groups #1, #2, #3, and #4, respectively (*P* = 0.256 and *P* = 0.138 for groups #1 vs. #2, and groups #3 vs. #4, respectively). While the proportions of participants with a MoCA score below 2 SD were 31.7%, 21.3%, 30.2%, and 24.4%, respectively (*P* = 0.334 and *P* = 0.146 for groups #1 vs. #2, and groups #3 vs. #4, respectively). Participants with a MoCA score below 22 (the cutoff for mild cognitive impairment) represented 52.5% and 57.5% of groups #1 and #2 (*P* = 0.493), and 31.0% and 19.4% (*P* = 0.003) of groups #3 and #4, respectively. Participants with a MoCA score below 17 (the cutoff for dementia) represented 23.8% and 16.1% of groups #1 and #2 (*P* = 0.192), and 8.3% and 4.7% (*P* = 0.091) of groups #3 and #4, respectively (Fig. 1).

### The prevalence of cognitive impairment

Of the 698 participants, 279 (40.0%) had an abnormal score in the MoCA and were invited for the neuropsychological assessment. This comprehensive cognitive evaluation was performed on 189 participants. The prevalence of cognitive impairment was 19.1% [95% CI 11.5%, 28.8%], 6.8% [95% CI 2.3%, 15.3%], 10.7% [95% CI 7.0%, 15.5%], and 3.2% [95% CI 1.3%, 6.4%], in groups #1, #2, #3, and #4, respectively. Hospitalized patients (groups #1 and #2) presented more frequently three or more impaired cognitive domains than the non-hospitalized participants (54.6% vs. 19.4%; *P* = 0.008) (Supplementary Fig. S6).

### Secondary analysis

Among participants with an abnormal MoCA score who did not perform the neuropsychological assessment, those in groups #1 and #3 more frequently reported cognitive complaints than those of the comparison groups (groups #2 and #4, respectively; Supplementary Table S5). Moreover, the predicted probabilities of having cognitive impairment were higher in group #1 than #2 (median [25th percentile, 75th percentile]: 20.0% [7.31%, 32.4%] vs. 4.7% [2.7%, 7.4%]; *P* = 0.017) and in group #3 than #4 (median [25th percentile, 75th percentile]: 7.1% [3.4%, 13.1%] vs. 1.9% [1.3%, 3.8%]; *P* < 0.001).

### Associations between participants’ characteristics and cognitive impairment

**Fig. 2:**
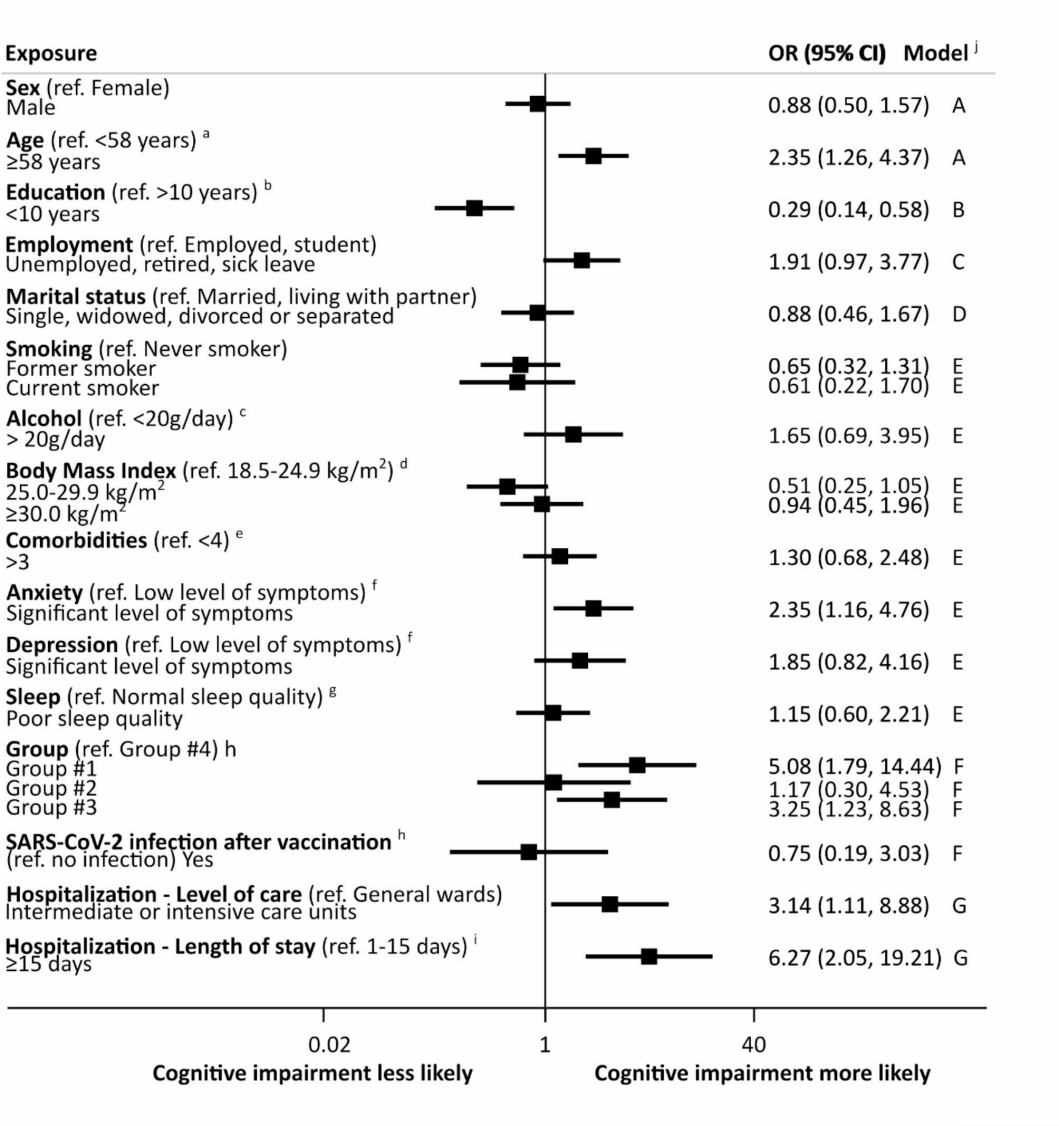
Associations between participants’ characteristics and cognitive impairment. *CI* confidence interval, *OR* odds ratio. ^a^Median age of all participants is 58 years. ^b^Median education of all participants is 10 years. ^c^Median number of comorbidities is 4. ^d^20 g/day is the threshold for acceptable consumption in males younger than 65 years; 10 g/day is the threshold for all females and males aged 65 or older. ^e^Body mass index categories of the World Health Organization. ^f^The cutoff of 11 in the Hospital Anxiety and Depression Scale anxiety/depression subscale was used to classify symptom levels. ^g^The cutoff of 5 in the Pittsburgh Sleep Quality Index scale was used to classify sleep quality. ^h^Participants grouped based on hospitalization and SARS-CoV-2 infection status between March 2020 and February 2021: group #1—hospitalized due to COVID-19; group #2—uninfected, hospitalized; group #3—infected, non-hospitalized; group #4—uninfected, non-hospitalized. ^i^Self-reported episode of COVID-19 after at least 14 days of complete primary scheme of vaccination (in groups #2 and #4). ^j^Logistic regression models A to G include the dependent outcome variable “cognitive impairment”, the independent variable “Exposure” and the following covariates, as potential confounders of the association between the exposure variable and the outcome variable: A—no covariates’ B—sex, age; C—sex, education, and comorbidities; D—sex and education; E—sex, age, and education; F—comorbidities, education, anxiety and depression symptoms, poor sleep, body mass index, employment, and smoking; G—sex, age, education, comorbidities.

Older age (≥ 58 years [median age]), lower education (< 10 years [median years of schooling]), clinically significant levels of anxiety, and SARS-CoV-2 infection were associated with higher odds of cognitive impairment (Fig. 2).

Compared to group #2, cognitive impairment was nearly five times higher in group #1 (adjusted OR 5.39, 95% CI 1.54, 18.92). The odds were three times higher in group #3 than in group #4 (adjusted OR 3.23, 95% CI 1.22, 8.57).

Cognitive impairment was more frequent among participants hospitalized in intermediate or intensive care units than in the general ward (adjusted OR 3.14, 95% CI 1.11, 8.88). Hospitalization for more than 15 days versus up to 15 days was associated with cognitive impairment (adjusted OR 6.27, 95% CI 2.05, 19.21) (Fig. 2).

Among participants infected with SARS-CoV-2 in any period of the pandemic (all groups), cognitive impairment was similarly frequent in participants infected once, twice, and thrice (9.5%, 9.4%, and 11.1%, respectively; *P* = 0.986). No statistically significant differences in cognitive impairment frequency were observed within group #4 between participants who reported never having been infected and those infected following COVID-19 vaccination (4.9% vs. 2.1%, respectively, *P* = 0.260). Similarly, in group #2, cognitive impairment was present in 7.0% and 6.8% of never infected versus infected (*P* = 0.959).

### Past and current symptoms and cognitive impairment

Among participants who tested positive for SARS-CoV-2 infection between March, 2020, and February, 2021, those with cognitive impairment after nearly 2 years reported more frequently having had vision changes (23.7% vs. 12.2%, *P* = 0.055), disorientation (39.5% vs. 11.8%, *P* < 0.001), changes in gait or balance (42.1% vs. 20.1%, *P* = 0.003), respiratory failure (26.3% vs. 14.2%, *P* = 0.057), and loss or decrease in appetite (60.5% vs. 42.5%, *P* = 0.037) during the acute phase of COVID-19. They also reported more frequently having felt dizziness after infection (26.8% vs. 11.5%, *P* = 0.007) and presented more frequently with the current symptoms: dizziness (22.0% vs. 9.6%, *P* = 0.019), disorientation (17.1% vs. 6.7%, *P* = 0.022), changes in gait or balance (31.7% vs. 12.5%, *P* = 0.001), myalgia (35.0% vs. 14.8%, *P* = 0.002), and oblivions (70.7% vs. 50.9%, *P* = 0.018) (Supplementary Tables S6–S8).

## Discussion

Cognitive impairment 2 years after SARS-CoV-2 infection in unvaccinated individuals affected nearly one-fifth of individuals hospitalized due to COVID-19 and one-tenth of non-hospitalized infected individuals. Survivors of COVID-19 of the first year of the pandemic were three to five times more likely to present cognitive impairment 2 years following SARS-CoV-2 infection than the general population.

As far as we know, this is the first study reporting the prevalence of cognitive impairment assessed with a neuropsychological battery of tests 2 years after SARS-CoV-2 infection in hospitalized and non-hospitalized COVID-19 cases. A previous study reported the persistence of cognitive complaints in people with post-COVID-19 condition 2 years after hospitalization for COVID-19 during the first wave of the pandemic. However, no cognitive tests were used to assess cognitive performance^23^. Another study included only patients hospitalized with COVID-19 between April and September 2020, who had a neurological consultation during their stay in the intensive care unit or general ward. The study reported cognitive impairment (a score in the Mini-Mental State Examination below 24) 2 years after hospitalization in 36% of participants who had an altered mental state during hospitalization, and in 17% of those with a normal mental state during hospitalization^24^. A systematic review and meta-analysis of studies published until June 2021 reported a pooled estimated prevalence of cognitive impairment (not self-reported) of 36% in survivors of COVID-19. Cognitive impairment was assessed almost exclusively with a screening instrument, namely the MoCA and different cutoffs were used, such as 18, 23, 26, or 1.5 SD of norms. No significant effect of time elapsed since SARS-CoV-2 infection (< 6 months vs. 6–12 months) was observed, but a higher prevalence among those hospitalized (30%) compared to non-hospitalized infected participants (20%) (*P* = 0.096) was reported^25^. A community-based study in China reported a prevalence of cognitive impairment (MoCA < 18) of 15.6% after a median time of 625 days from SARS-CoV-2 infection^26^. This result is similar to ours: 14.7% of groups #1 and #3 have a MoCA score < 18. However, in previous studies, the absence of a comparison group made the prevalence of cognitive impairment challenging to apprise. Moreover, a single assessment with a screening test such as the MoCA limits the accuracy of identifying cognitive impairment. We used a two-step approach, using the MoCA to screen the whole sample, and the neuropsychological assessment to confirm cognitive impairment. This improved the validity of our results. In the present study, participants presented a wide range of education levels, with almost half of hospitalized participants (groups #1 and #2) having only primary education. This may explain the divergent result (no association of hospitalization for COVID-19 with cognitive impairment) with another study^6^.

Evidence prior to the COVID-19 pandemic suggests that episodes of hospitalization affect cognitive performance in elderly patients^27^. Length of stay, and reduced mobility and activities during hospitalization were associated with cognitive decline in patients with a bone fracture^28^. Delirium, use of corticosteroids, stress, and depression were identified as possible mediators in the association between hospitalization and cognitive impairment^29^. Moreover, non-elective hospitalization appears to be associated with a faster cognitive decline in older persons^30^. In the present study, hospitalization in intensive or intermediate care units, more than 15 days of hospitalization, disorientation, vision changes, changes in gait or balance, and loss of appetite during infection were associated with cognitive impairment. This information could help in the identification of survivors of COVID-19 more likely to have cognitive impairment, and for whom special clinical attention may improve the timely diagnosis of a cognitive disorder and its treatment.

Almost 60% of hospitalized participants (groups #1 and #2) with cognitive impairment had a multidomain impairment, with four to five domains affected out of five. This suggests that not only is cognitive impairment more frequent, but it is also more severe in hospitalized patients. The higher frequency and severity of cognitive impairment in SARS-CoV-2 infected patients—hospitalized versus non-hospitalized—may not have been identified in studies using screening tests^25^, as in our study, the prevalence of abnormal MoCA scores was similar between hospitalized and non-hospitalized COVID-19 survivors.

Our results suggest that infection with SARS-CoV-2 after primary vaccination is not associated with a higher frequency of cognitive impairment. However, the study was not designed for this purpose and may not include a sufficient number of never infected participants to detect a possible lower effect size. Vaccination against COVID-19 has an approximately 30% efficacy in preventing long-COVID^31^. The prevalence of long-COVID was reported to be lower in the eras of the delta and omicron variants^32,33^. This suggests that vaccination status and the virus variant influence the outcomes of SARS-CoV-2 infection, which may include an effect on cognitive performance. It is increasingly difficult to conduct a study comparing individuals infected with SARS-CoV-2 with those who have never been infected, as the second group is increasingly smaller.

This study has limitations that should be acknowledged. First, the observational cross-sectional design with no pre-pandemic data on cognitive impairment prevents the establishment of causality between SARS-CoV-2 infection and cognitive impairment. Participants with a previous diagnosis of dementia or another neurologic or psychiatric condition impairing cognitive performance were excluded from the study. Still, we did not assess the presence of undiagnosed cognitive impairment before SARS-CoV-2 infection or hospitalization. Therefore, a direct causal effect of SARS-CoV-2 infection on cognitive impairment cannot be inferred from our results. This is a limitation of most studies on COVID-19 and cognitive dysfunction, as pre-pandemic data are usually unknown due to the lack of routine cognitive assessments. However, a cohort study regularly evaluating cognitive performance before and during the pandemic reported an association between mild COVID-19 and cognitive decline^34^. The effect of SARS-CoV-2 infection in the brain could be explained by different mechanisms to which virus and host characteristics would contribute alongside the pathway from virus entrance, pathologic responses, neuroinvasion, and neurovirulence^35^. Genome-wide association studies have identified genes involved in the pathogenicity of both COVID-19 and Alzheimer´s disease, namely the ε4 allele of the APOE gene. These could explain the higher risk of Alzheimer’s disease after hospitalization for COVID-19^36,37^.

A second limitation of this study relies on the ascertainment of SARS-CoV-2 infection. Indeed, undiagnosed exposure to SARS-CoV-2 infection before vaccination was not assessed in participants of the comparison groups (groups #2 and #4) as serological tests were not performed. Nevertheless, we may expect a low rate of misclassification considering data on seroprevalence for COVID-19 and vaccination uptake. Indeed, the National Serological Survey reported a low seroprevalence (2.9%) in May–June 2020^38^, although higher, 15.5%, in February–March 2021 (13.5% due to natural infection)^39^. Self-testing for COVID-19 became available in March 2021, and mass vaccination of the population reached 73% and 83% with one and two doses, respectively, on August 28, 2021, and 86% with doses on October 31, 2021^40^.

Third, a third of participants eligible for the neuropsychological assessment (MoCA score below 1.5 SD of age- and education-specific mean of normative data) did not perform this evaluation, with almost half declining to participate. Reasons for refusal could be linked with a higher or lower likelihood of objective cognitive impairment. On the one hand, a participant, aware of some cognitive changes, is afraid of confirmation of cognitive impairment and refuses to be evaluated. On the other hand, a participant refuses the neuropsychological assessment, believing it is unnecessary due to a perceived lack of cognitive changes. As cognitive complaints were more frequent in eligible non-assessed participants of groups #1 versus #2 and groups #3 versus #4, we may expect that the prevalence of cognitive impairment could be even higher in the groups of infected individuals (groups #1 and #3) than in the comparison groups (groups #2 and #4, respectively). It should be noted that a person with cognitive impairment may not be aware of their own cognitive decline although noticed by the family^41^, and cognitive complaints are frequently reported in middle-aged and older individuals with normal results in cognitive testing^42^. We also computed the predicted probability of cognitive impairment among these eligible non-assessed participants, and the association between SARS-CoV-2 infection and cognitive dysfunction was still positive and significant.

Lastly, although we considered the most relevant confounders, other characteristics such as practice of physical activity, leisure activities, social isolation, and occupation may have contributed for residual confounding, with an unpredictable impact on the results. Furthermore, we could only include a few participants admitted to intermediate and intensive care units due to hospital capacity, number of patients requiring critical care, and deaths before recruitment. Moreover, elective surgeries were postponed during the first wave of the pandemic and progressively scheduled afterwards. This may explain our difficulties in recruiting eligible participants for group #2. The low number of participants admitted to intermediate and intensive care units did not allow us to adequately study the contribution of level of hospital care, a proxy of disease severity, as a mediator or moderator factor in the association between SARS-CoV-2 infection and cognitive impairment. Nevertheless, the strength of association was reduced in non-hospitalized participants. The consumption of some medicines, such as those with anticholinergic activity, opioid agents, psychotropic drugs, immunosuppressive drugs, but also some antibacterial and antiviral agents have been reported to have negative effects on cognitive function^43^. We did not observe a higher current consumption of drugs acting in the central nervous system in the groups infected with SARS-CoV-2 (groups #1 and #3) and we did not collect information on their use before SARS-CoV-2 infection. However, based on the existing literature, it is unlikely that these drugs could be risk factors of SARS-CoV-2 infection, and therefore, confounders of the association between SARS-CoV-2 infection and cognitive impairment. A possible association between SARS-CoV-2 infection and the consumption of these drugs would rather be in the sense of the consumption of these drugs because of COVID-19. The contribution of this possible mediator effect should be investigated in future studies. The cognitive assessment of participants of the NeurodegCoV-19 project after 1 year and the clinical assessment by a neurologist is ongoing and will help clarify the contribution of possible factors for cognitive impairment.

## Conclusions

Infection with SARS-CoV-2 during the first year of the pandemic was associated with three to five times higher odds of cognitive impairment 2 years after infection. Survivors of the disease may require special attention from clinical doctors to diagnose and treat cognitive impairment, namely, those who were hospitalized for more than 15 days, in intermediate or intensive care units, and presented disorientation, changes in vision, gait or balance, during infection. Conversely, although cognitive impairment was less frequent and severe in COVID-19 cases who had been followed in the community during infection, this population is younger. Causality between SARS-CoV-2 infection and cognitive impairment could not be inferred from the present study. The follow-up of participants of the NeurodegCoV-19 and other COVID-19 cohorts are needed to better understand the long-term relation between COVID-19 and cognitive dysfunction. Nevertheless, in clinical practice, this antecedent may constitute as relevant information among other characteristics of the patients presenting a possible neurodegenerative cognitive dysfunction.

## Electronic supplementary material

Below is the link to the electronic supplementary material.

Supplementary Material 1

## Data Availability

Requests for accessing individual de-identified participant data can be sent to the corresponding author. They will be analyzed by the research team, the Ethics Committee and the Data Protection team of the research institution hosting the study, the Public Health Institute of the University of Porto, to ensure that the data will be used only for research purposes, aligned with the research objective of the study to which participants agreed, as well as ensuring data protection.
