# Supplementary material for: Cognitive impairment 2 years after mild to severe SARS-CoV-2 infection in a population-based study with matched-comparison groups
Source: Sci Rep. 2025 Jul 8;15:24335. doi: 10.1038/s41598-025-96608-0 (PMC12234903; doi:10.1038/s41598-025-96608-0)
Supplement: Supplementary file 1 — Supplementary Material 1 [file 41598_2025_96608_MOESM1_ESM.pdf]

Cognitive impairment two years after mild to severe SARS-CoV-2 infection: a population-based study with matched-comparison groups. Natália Araújo, Isa Silva, Patrícia Campos, Adriana Costa, Catarina Lopes, Mariana Seco, Ana Rute Costa, Maria Margarida Calejo, Maria Joana Pais, Susana Pereira, Samantha Morais, João Firmino Machado, Luís Ruano, Nuno Lunet, Vítor Tedim Cruz.

Supplementary material

Table S1. Eligibility criteria for participation. 2

Table S2. Number of SARS-CoV-2 infections and period of first positive test, from March 2020 to the baseline evaluation (July 2022-October 2023) in participants grouped according to hospitalization and positivity for SARS-CoV-2 infection between March 2020 and February 2021. 4

Table S4. Potential confounders in the association between SARS-CoV-2 infection and cognitive impairment. 6

Table S5. The relative frequency of participants who reported cognitive complaints among participants with a Montreal Cognitive Assessment score below age- and education-specific cut-offs who were not evaluated with the neuropsychological battery of tests. 7

Table S6. Characteristics of participants with and without cognitive impairment. 8

Table S7. Frequency of symptoms during SARS-CoV-2 infection in participants infected with SARS-CoV-2 between March 2020 and February 2021, with and without cognitive impairment. 10

Table S8. Frequency of symptoms after SARS-CoV-2 infection in participants infected with SARS-CoV-2 between March 2020 and February 2021, with and without cognitive impairment. 11

Table S9. Frequency of current symptoms in participants infected with SARS-CoV-2 between March 2020 and February 2021, with and without cognitive impairment. 12

Figure S1. Identification of participants from the lists of diagnostic (RT-PCR) test results for SARS-CoV-2 infection and hospital admissions at Hospital Pedro Hispano (reference hospital of the Local Health Unit of Matosinhos) in the period from March 2020 to February 2021. 14

Figure S2. Selection of participants for group #1. 15

Figure S3. Selection of participants for group #2. 16

Figure S5. Selection of participants for group #4. 18

Figure S6. Impaired cognitive domains in each group of participants: **A)** Percentages of participants presenting 1, 2, 3, 4, and 5 impaired cognitive domains in each group; **B)** percentages of participants in each group presenting impairment in the cognitive domains assessed with the neuropsychological battery of tests. 19

Table S1. Eligibility criteria for participation.

Study group	Inclusion criteria	Exclusion criteria
All groups	Enrollment in the Local Health Unit of Matosinhos (ULSM), which covers nearly all residents of Matosinhos municipality, that is, individuals with a unique 9-digit number within the Portuguese Health System and enrolled at a primary care center of ULSM, according with administrative list.	Death;<br>Living in a nursing home;<br>Physical (motor, visual or auditory problem) or medical (eg.: under chemotherapy treatment) limitation for participating;<br>Neurological or psychiatric conditions deemed by the neurologist to affect cognitive function;<br>Consumption of anti-dementia drugs – Memantine, Rivastigmine, or Donepezil;<br>Participation in psychotherapeutic interventions to prevent or control cognitive decline (clinical treatment or research study);<br>Being under clinical neuropsychological assessment;<br>History of substance abuse (registered in the medical file or spontaneously reported by the participant);<br>Illiterate;<br>Less than one year of formal education;<br>Primary education (grades one to four) not in the Portuguese Education System;<br>Unable to understand the study;<br>Residency not in the municipality of Matosinhos.
Goup #1	With a positive result in the list of all diagnostic tests for COVID-19 (Real-Time Polymerase Chain Reaction on samples of nasopharyngeal or oropharyngeal swabs) registered at the ULSM database between March 2020 and February 2021;<br>Age equal to or above 18 years at SARS-CoV-2 infection;<br>Admission at Hospital Pedro Hispano (reference hospital of the ULSM) due to SARS-CoV-2 infection (acute phase of COVID-19) between March 2020 and February 2021, confirmed by	Admission at a private hospital for SARS-CoV-2 infection (acute phase of COVID-19);<br>SARS-CoV-2 infection after a first dose of COVID-19 vaccination.

	consultation of electronic health record and reports from participants.	
Group #2	Age equal to or above 18 years at hospital admission;<br>With an episode of hospital admission at Hospital Pedro Hispano between March 2020 to February 2021, registered in the administrative list of ULSM;<br>With complete primary vaccination for COVID-19 (self-reported).	With a positive test in the list of all diagnostic tests for COVID-19 (Real-Time Polymerase Chain Reaction on samples of nasopharyngeal or oropharyngeal swabs) registered at the ULSM database between March 2020 to February 2021;<br>SARS-CoV-2 infection (self-reported) in the period from March 2020 to February 2021;<br>SARS-CoV-2 infection (self-reported) between the two doses of the primary vaccination scheme or within the 14 days following vaccination.
Group #3	With a positive test in the list of all diagnostic tests for COVID-19 (Real-Time Polymerase Chain Reaction on samples of nasopharyngeal or oropharyngeal swabs) registered at the ULSM database between March 2020 to February 2021;<br>Age equal to or above 18 years at SARS-CoV-2 infection.	SARS-CoV-2 infection after a first dose of COVID-19 vaccination;<br>Hospitalization at any hospital for SARS-CoV-2 infection between March 2020 to February 2021.
Group #4	Age above 18 years at recruitment;<br>With complete primary vaccination for COVID-19 (self-reported).	With a positive test in the list of all diagnostic tests for COVID-19 (Real-Time Polymerase Chain Reaction on samples of nasopharyngeal or oropharyngeal swabs) registered at the ULSM database between March 2020 to February 2021;<br>SARS-CoV-2 infection (self-reported) in the period from March 2020 to February 2021;<br>SARS-CoV-2 infection (self-reported) between the two doses of the primary vaccination scheme or within the 14 days following vaccination;<br>Hospitalization at any hospital between March 2020 to February 2021.

Table S2. Number of SARS-CoV-2 infections and period of first positive test, from March 2020 to the baseline evaluation (July 2022-October 2023) in participants grouped according to hospitalization and positivity for SARS-CoV-2 infection between March 2020 and February 2021.

	Hospitalized		Non-hospitalized	
	Due to COVID-19	Without a positive test for SARS-CoV-2 infection	With a positive test for SARS-CoV-2 infection	Without a positive test for SARS-CoV-2 infection
	Group #1<br>n (%)	Group #2<br>n (%)	Group #3<br>n (%)	Group #4<br>n (%)
Number of infections				
0	0 (0.0)	52 (60.5)	0 (0.0)	93 (36.1)
1	81 (80.2)	32 (37.2)	176 (69.8)	161 (62.4)
2	18 (17.8)	2 (2.3)	69 (27.4)	2 (0.8)
3	2 (2.0)	0 (0.0)	7 (2.8)	2 (0.8)
Period of infection				
Mar. – Aug. 2020	13 (12.9)	-	34 (13.4)	-
Oct. –Dec. 2020	59 (58.4)	-	173 (68.7)	-
Jan.– Feb. 2021	29 (28.7)	-	45 (17.9)	-
Mar. – May 2021	-	2 (5.9)	-	0 (0.0)
June – Dec. 2021	-	6 (17.7)	-	12 (7.3)
After January 2022	-	26 (76.5)	-	153 (92.7)

Table S3. Criteria for the definition of impaired cognitive domain.

**Cognitive domain**	**Test**	**Criteria for impairment**
**Verbal memory**	WMS III – Logical Memory I and II	At least two scores < 1.5 SD or 3 scores < 1 SD
**Visual memory**	WMS III – Visual Reproduction I and II	At least two scores < 1.5 SD or 3 scores < 1 SD
**Attention/Information processing speed**	WAIS-III – Digit – Symbol – Coding and Symbol search	At least two scores < 1.5 SD or 3 scores < 1 SD
	Trail Making Test, part A	
	Stroop test – word reading	
**Executive functions**	Stroop test (color naming and word color naming)	At least three scores < 1.5 SD or 2 scores < 2 SD
	Trail Making Test, part B, and B-A	
	Phonemic Fluency – letters M, R, and P	
	Phonemic Fluency – categories of animals	
	WMS III– Digit span	
**Language**	Token Test – short-form	Score <2 SD

SD, standard deviation; WAIS-III, Wechsler Adult Intelligence Scale – Third Edition; WMS-III, Wechsler Memory Scale – Third Edition.

Table S4. Potential confounders in the association between SARS-CoV-2 infection and cognitive impairment.

Risk factors for SARS-CoV-2 infection	Risk factors for cognitive impairment	Variable	Not assessed/Reason for non-assessment
Minority ethnicity/race/country of birth	✓	Not Assessed	Exclusion criteria for applying cognitive tests
Lower socioeconomic status	✓	Education was used as a proxy	
Older age	✓	Age	
Increased household size/cohabitants and type of housing	✓ (social isolation)	Not Assessed	Only current marital status was assessed
Presence of comorbidities/health status	✓	A list of comorbidities; self-rated health status; Medicines consumption	
Male sex	✓ (for some types of cognitive impairment)	Sex	
Occupation	✓ (Unemployed/retired/sick leave)	Current situation (unemployed/retired/on sick leave)	
Lower income level/occupation status	✓	Education used as a proxy	
Obesity	✓	Current body mass index categories; previous diagnosis of obesity	
Lower education level	✓	Years of schooling	
Residential factors - rural/urban, population size	✓	Not assessed	Slight variation within the municipality of Matosinhos
Employment factors - inability to work from home	✗		
Presence of mental disorders/psychiatric diagnosis	✓	Current anxiety and depression symptoms, and medicines consumption; previous diagnoses of mental disorders	
Environmental - lack of access to safe drinking water, increased air pollution	Exposure to metals and air pollution?	Not assessed	
Smoking status	✓	Current and past smoking status	
Alcohol consumption	✓	Current alcohol consumption	

Table S5. The relative frequency of participants who reported cognitive complaints among participants with a Montreal Cognitive Assessment score below age- and education-specific cut-offs who were not evaluated with the neuropsychological battery of tests.

	Group #1 %	Group #2 %	P value	Group #3 %	Group #4 %	P value
Forgetfulness	58.3	7.1	0.005	32.1	36.1	0.740
Slowing down of thinking	41.7	0.0	0.007	21.4	11.1	0.259
Distraction	8.3	14.3	0.636	25.0	5.6	0.026

Table S6. Characteristics of participants with and without cognitive impairment.

	Cognitive impairment		
	No<br>n (%)	Yes<br>n (%)	P-value
Sex			0.665
Female	318 (90.9)	32 (9.1)	
Male	237 (91.9)	21 (8.1)	
Age (years) [a]			0.006
<58	267 (94.7)	15 (5.3)	
≥58	288 (88.3)	38 (11.7)	
Education (years) [b]			<0.001
<10	254 (86.4)	40 (13.6)	
≥10	301 (95.9)	13 (4.1)	
Employment			<0.001
Employed	329 (95.1)	17 (4.9)	
Unemployed, retired, on sick leave	226 (86.6)	35 (13.4)	
Marital status [c]			0.048
Single	69 (98.6)	1 (1.4)	
Married/living with a partner	379 (90.9)	38 (9.1)	
Widowed, divorced, separated	106 (88.3)	14 (11.7)	
Smoking			0.183
Never smoker	276 (89.3)	33 (10.7)	
Ex-smoker	189 (92.6)	15 (7.4)	
Current smoker	90 (94.7)	5 (5.3)	
Alcohol consumption [d]			0.096
≤20 g/day	509 (91.9)	45 (8.1)	
>20 g/day	46 (85.2)	8 (14.8)	
Body mass index (kg/m^2) [d]			0.162
<18.5	7 (100.0)	0 (0.0)	
18.5-24.9	180 (90.9)	18 (9.1)	
25.0-29.9	243 (93.5)	17 (6.5)	
≥30.0	123 (87.2)	18 (12.8)	
Co-morbidities [e]			0.013
<4	308 (93.9)	20 (6.1)	
≥4	247 (88.2)	33 (11.8)	
Anxiety symptoms [f]			0.004
Normal levels	460 (92.9)	35 (7.1)	
Clinically significant levels	85 (84.2)	16 (15.8)	
Depression symptoms [g]			0.064
Normal levels	492 (91.8)	44 (8.2)	
Clinically significant levels	49 (84.5)	9 (15.5)	
Sleep quality [h]			0.445
Normal	201 (92.6)	16 (7.4)	
Poor quality of sleep	305 (90.8)	31 (9.2)	
Group [i]			<0.001
#1	72 (80.9)	17 (19.1)	
#2	68 (93.2)	5 (6.8)	
#3	200 (89.3)	24 (10.7)	
#4	215 (96.8)	7 (3.2)	
SARS-CoV2 infection after vaccination in group #2 [j]			0.959
No	40 (93.0)	3 (7.0)	
Yes	28 (93.3)	2 (6.7)	

SARS-CoV2 infection after vaccination in group #4 [j]			0.260
No	78 (95.1)	4 (4.9)	
Yes	137 (97.9)	3 (2.1)	
Length of stay			<0.001
<15	112 (91.8)	10 (8.2)	
≥15 days	28 (70.0)	12 (30.0)	
Level of care			0.102
General ward	95 (89.6)	11 (10.4)	
Intermediate and intensive care units	45 (80.4)	11 (19.6)	

[a] Median age of all participants is 58 years

[b] Median education of all participants is 10 years

[c] 20 g/day is the threshold for men younger than 65 years; 10 g/day is the threshold for women and men aged 65 or older

[d] Body mass index categories of the World Health Organization

[e] Median number of comorbidities is 4

[f] The cutoff of 11 in the Hospital Anxiety and Depression – anxiety subscale – was used to classify symptoms levels

[g] The cutoff of 11 in the Hospital Anxiety and Depression – depression subscale – was used to classify symptoms levels

[h] The cutoff of 5 in the Pittsburgh Sleep Quality Index scale was used to classify sleep quality

[i] Participants grouped based on hospitalization and SARS-CoV-2 infection status between March 2020 and February 2021: group #1 – hospitalized due to COVID19; group #2 – uninfected, hospitalized; group #3 – infected non-hospitalized; group #4 – uninfected, non-hospitalized

[j] Self-reported episode of COVID-19 after at least 14 days of complete primary scheme of vaccination (in groups #2 and #4)

Table S7. Frequency of symptoms during SARS-CoV-2 infection in participants infected with SARS-CoV-2 between March 2020 and February 2021, with and without cognitive impairment.

	Cognitive impairment				
	No		Yes		
	n	%	n	%	P value
Headaches (cephalalgias)	135	53.1	20	52.6	0.952
Loss of smell (hyposmia/anosmia)	137	53.9	20	52.6	0.880
Loss or changes in taste (hypogeusia/dysgeusia)	143	56.3	19	50.0	0.466
Vision changes	31	12.2	9	23.7	0.055
Disorientation	30	11.8	15	39.5	**<0.001**
Delirium	20	7.9	4	10.5	0.579
Change in gait or balance	51	20.1	16	42.1	**0.003**
Temperature above 37.5ºC	128	50.4	24	63.2	0.142
Cough	143	56.3	23	60.5	0.624
Stuffy nose (nasal congestion)	100	39.4	15	39.5	0.990
Sore throat (odynophagia)	80	31.6	12	31.6	0.996
Chest (thoracic) pain	57	22.4	7	18.4	0.576
Difficulty breathing (dyspnea)	97	38.2	16	42.1	0.644
Respiratory failure	36	14.2	10	26.3	0.057
Loss or decrease in appetite (anorexia)	108	42.5	23	60.5	**0.037**
Diarrhea	61	24.0	8	21.1	0.688
Abdominal pain	24	9.4	2	5.3	0.398
Nausea/vomiting	46	18.1	9	23.7	0.412
Muscle pain throughout the body (myalgia)	158	62.2	24	63.2	0.910
More tired than usual	178	70.1	26	68.4	0.835

Table S8. Frequency of symptoms after SARS-CoV-2 infection in participants infected with SARS-CoV-2 between March 2020 and February 2021, with and without cognitive impairment.

	Cognitive impairment				
	No		Yes		
	n	%	n	%	P value
Headaches (cephalalgias)	59	21.9	10	24.4	0.715
Vision changes	42	15.6	7	17.1	0.804
Dizziness	31	11.5	11	26.8	**0.007**
Delirium	3	1.1	1	2.4	0.482
Excessive daytime sleepiness	77	28.6	10	24.4	0.574
Difficulty falling asleep	83	30.7	17	41.5	0.171
Difficulty staying asleep	92	34.1	15	36.6	0.752
Waking up too early	53	19.6	10	24.4	0.480
Another sleep problem	3	1.1	1	2.4	0.482
Oblivions	136	50.4	25	62.5	0.152
Slowing of thought	95	35.2	13	32.5	0.739
Distraction	84	31.1	14	35.0	0.622
Another cognitive complaint	3	1.1	0	0.0	0.503
Coma	2	0.7	0	0.0	0.584
Stroke	1	0.4	0	0.0	0.699
Epileptic seizures	2	0.7	1	2.6	0.278
Muscle problems (e.g., weakness, atrophy, cramps)	79	29.3	16	40.0	0.169
Loss of movement, sensation, or function in a specific location on the body, such as the left side of the face, left arm, or other areas	14	5.2	4	10.0	0.224

Table S9. Frequency of current symptoms in participants infected with SARS-CoV-2 between March 2020 and February 2021, with and without cognitive impairment.

	Cognitive impairment				
	No		Yes		
	n	%	n	%	P value
Headaches (cephalalgias)	69	25.5	10	24.4	0.883
Loss of smell (hyposmia/anosmia)	31	11.4	4	9.8	0.750
Loss or changes in taste (hypogeusia/dysgeusia)	27	10.0	4	9.8	0.967
Vision changes	54	19.9	9	22.0	0.763
Dizziness	26	9.6	9	22.0	**0.019**
Disorientation	18	6.7	7	17.1	**0.022**
Delirium	0	0.0	0	0.0	na.
Change in gait or balance	34	12.5	13	31.7	**0.001**
Temperature above 37.5ºC	1	0.4	0	0.0	0.697
Cough	28	10.3	2	4.9	0.272
Stuffy nose (nasal congestion)	35	12.9	5	12.2	0.904
Sore throat (odynophagia)	10	3.7	1	2.4	0.686
Chest (thoracic) pain	16	5.9	4	9.8	0.348
Difficulty breathing (dyspnea)	34	12.5	5	12.2	0.949
Respiratory failure	3	1.1	0	0.0	0.498
Loss or decrease in appetite (anorexia)	14	5.2	4	9.8	0.240
Diarrhea	15	5.5	0	0.0	0.123
Abdominal pain	10	3.7	0	0.0	0.211
Nausea/vomiting	8	3.0	2	4.9	0.514
Muscle pain throughout the body (myalgia)	40	14.8	14	35.0	**0.002**
More tired than usual	112	41.3	21	51.2	0.233
Excessive daytime sleepiness	53	19.6	11	26.8	0.282
Difficulty falling asleep	71	26.2	14	34.1	0.287

Difficulty staying asleep	87	32.1	16	39.0	0.380
Waking up too early	52	19.2	11	26.8	0.256
Another sleep problem	2	0.7	1	2.4	0.300
Oblivions	138	50.9	29	70.7	**0.018**
Slowing of thought	84	31.0	16	39.0	0.305
Distraction	72	26.6	15	36.6	0.183
Another cognitive complaint	3	1.1	0	0.0	0.498
Epileptic seizures	0	0.0	0	0.0	na.
Muscle problems (e.g., weakness, atrophy, cramps)	56	20.7	14	34.1	0.054
Loss of movement, sensation, or function in a specific location on the body, such as the left side of the face, left arm, or other areas	9	3.3	1	2.4	0.762

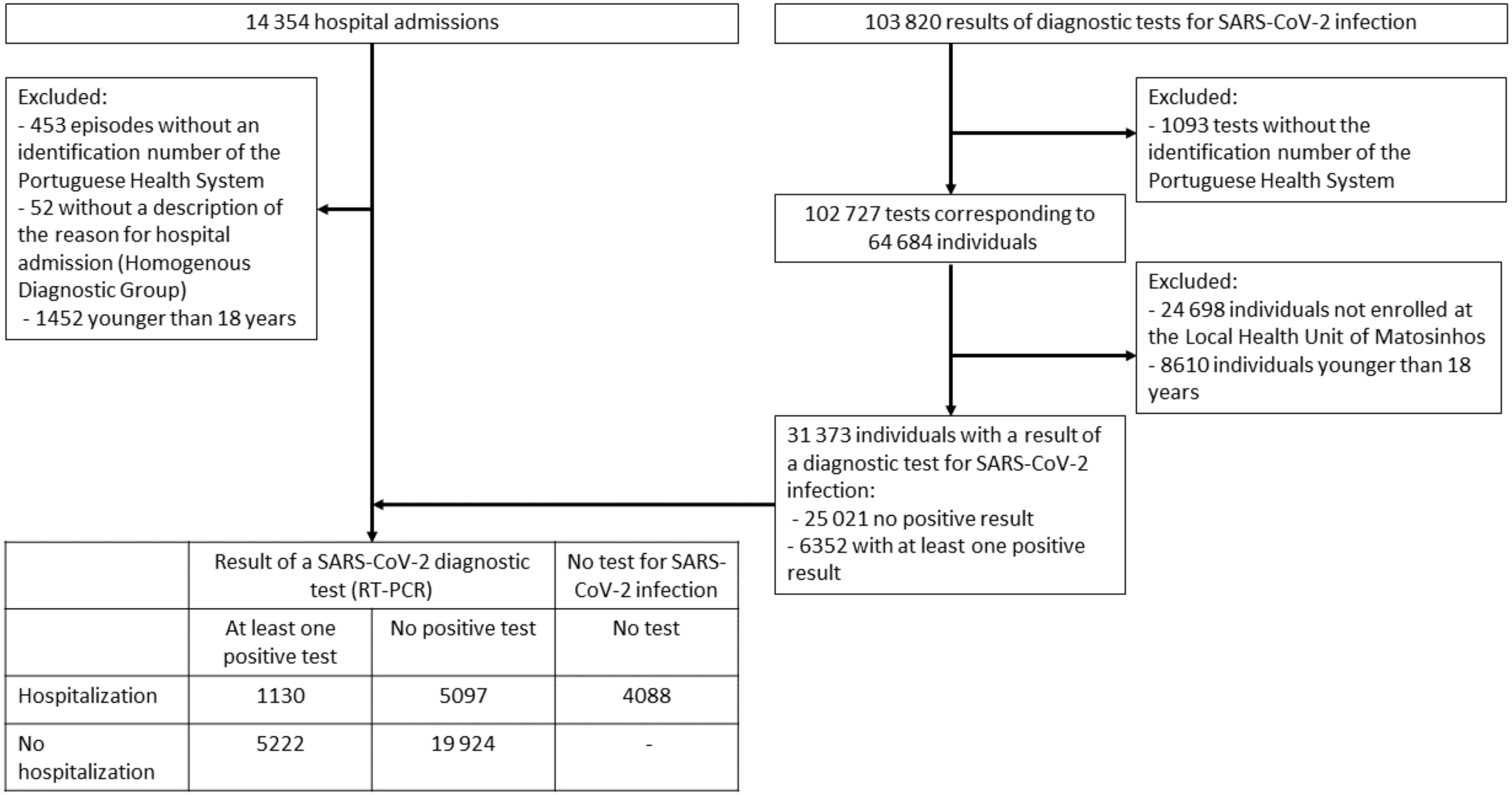

	Result of a SARS-CoV-2 diagnostic test (RT-PCR)		No test for SARS-CoV-2 infection
	At least one positive test	No positive test	No test
Hospitalization	1130	5097	4088
No hospitalization	5222	19 924	-

Figure S1. Identification of participants from the lists of diagnostic (RT-PCR) test results for SARS-CoV-2 infection and hospital admissions at Hospital Pedro Hispano (reference hospital of the Local Health Unit of Matosinhos) in the period from March 2020 to February 2021.

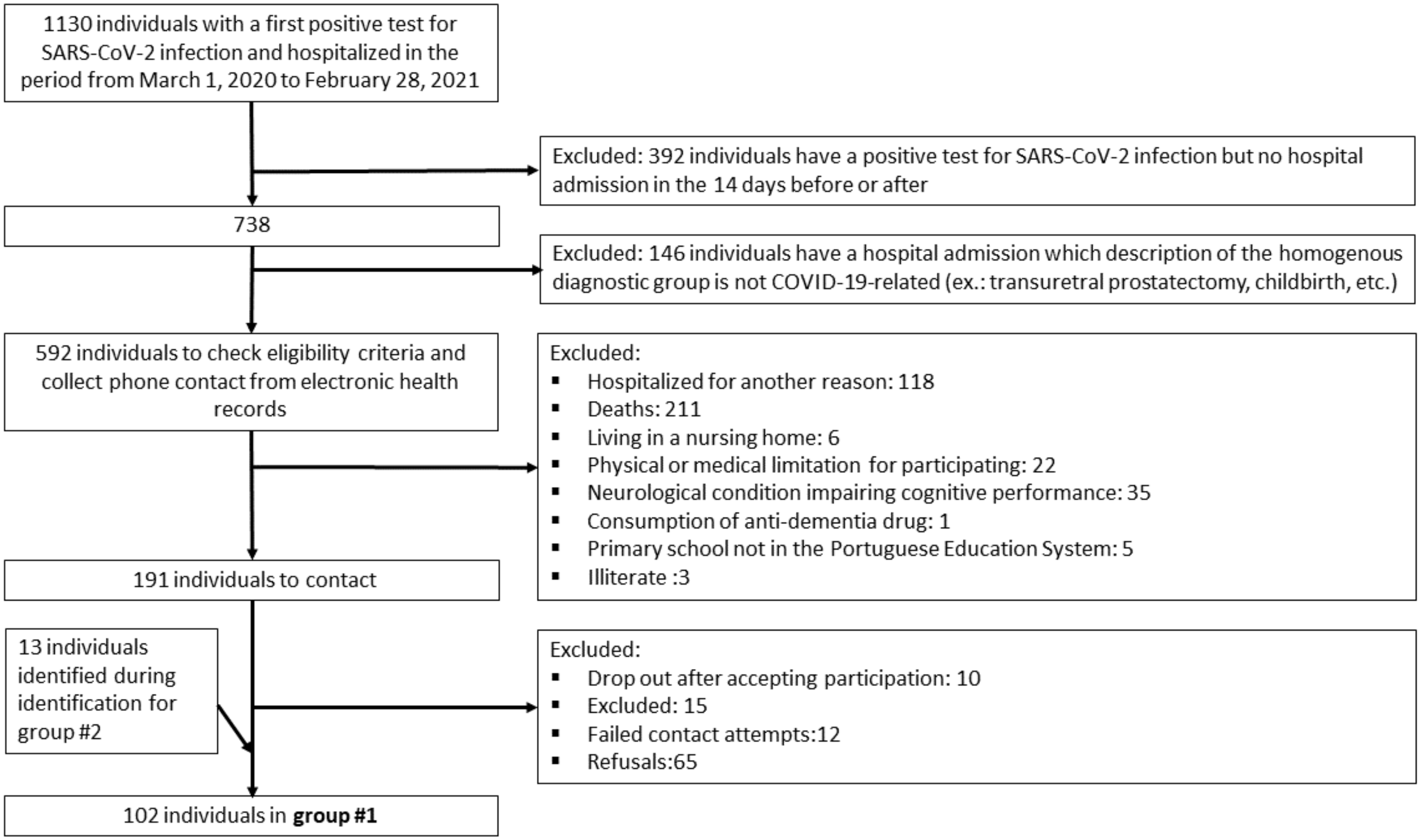

Figure S2. Selection of participants for group #1.

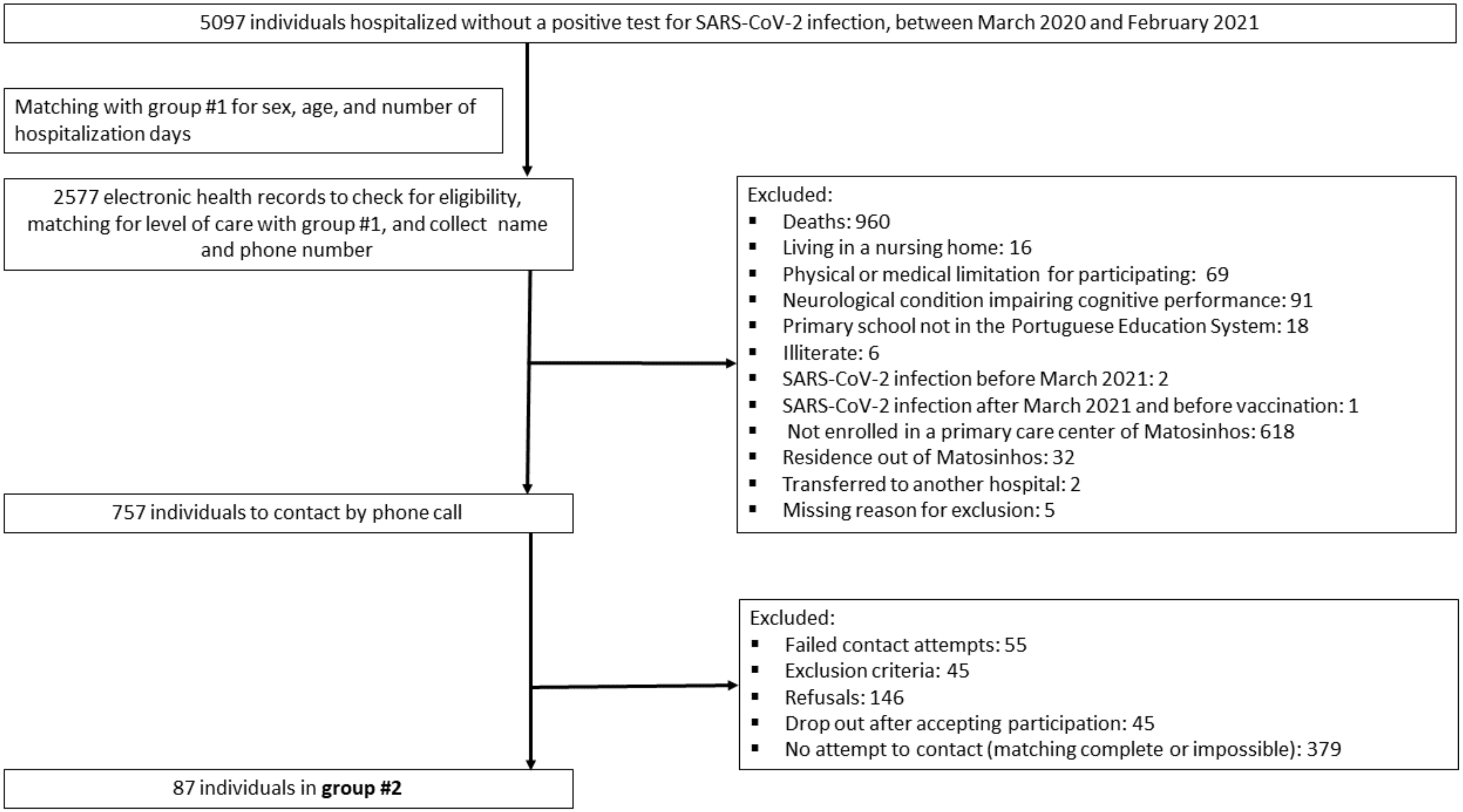

Figure S3. Selection of participants for group #2.

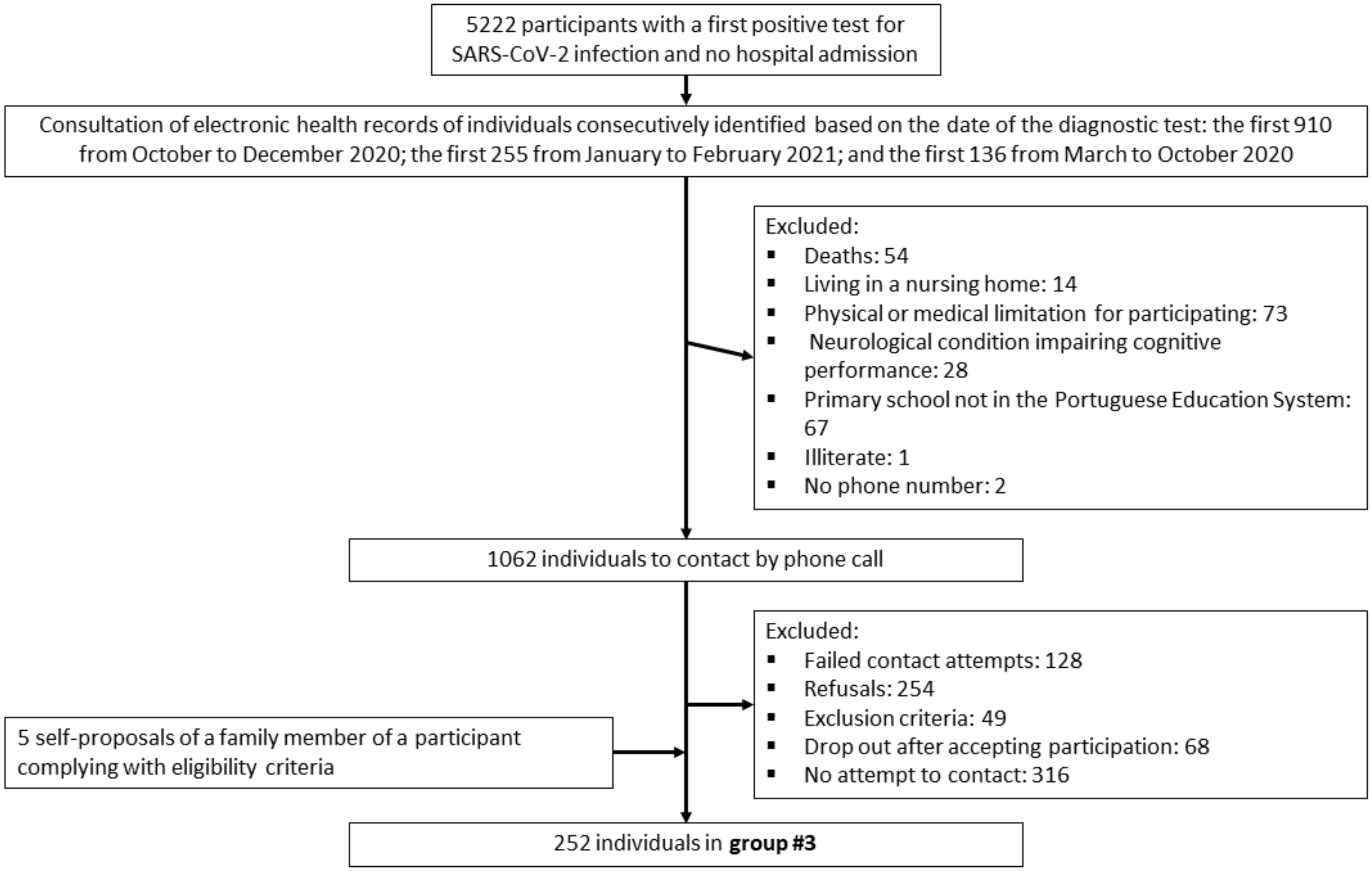

Figure S4. Selection of participants for group #3.

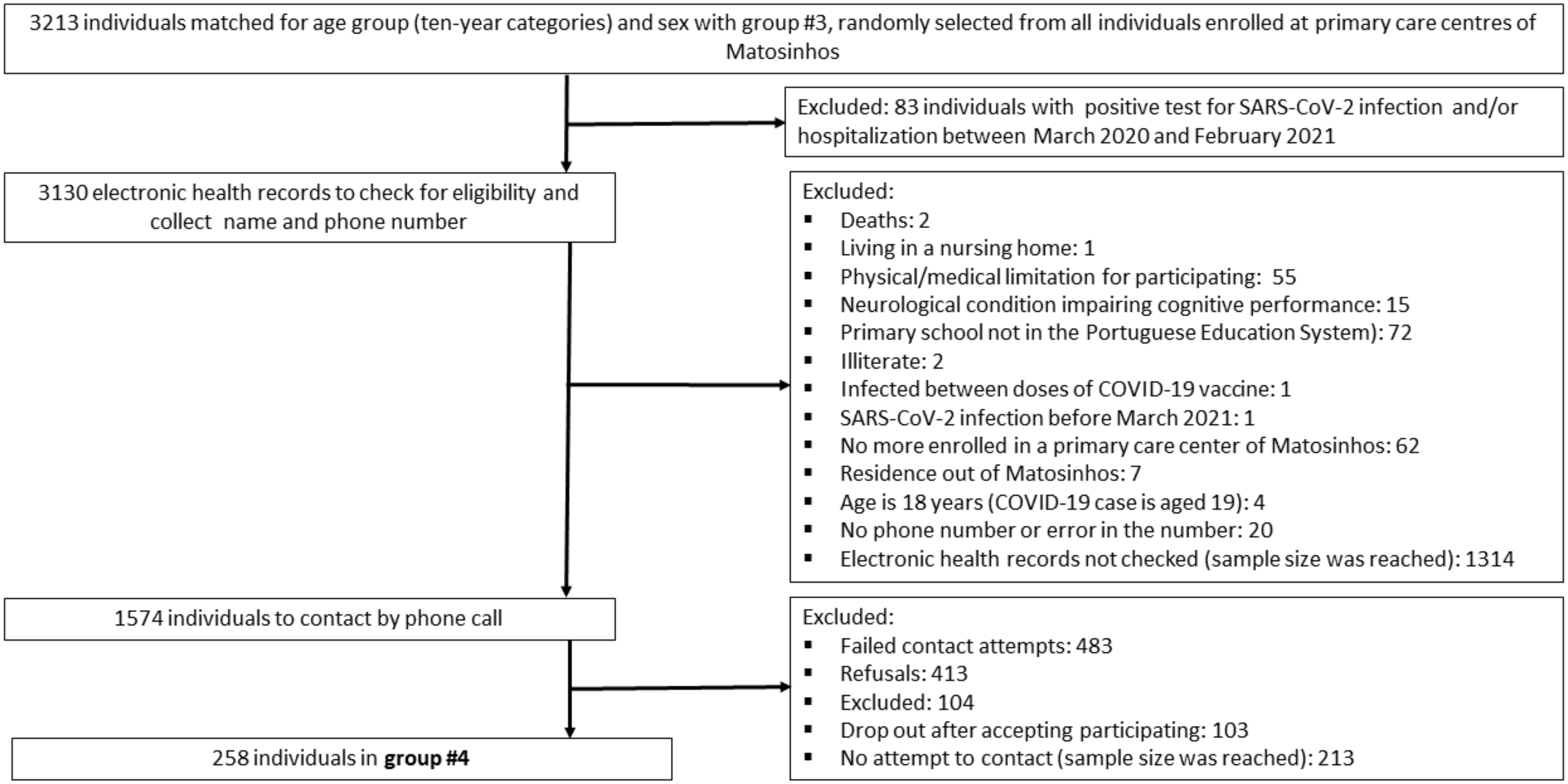

Figure S5. Selection of participants for group #4.

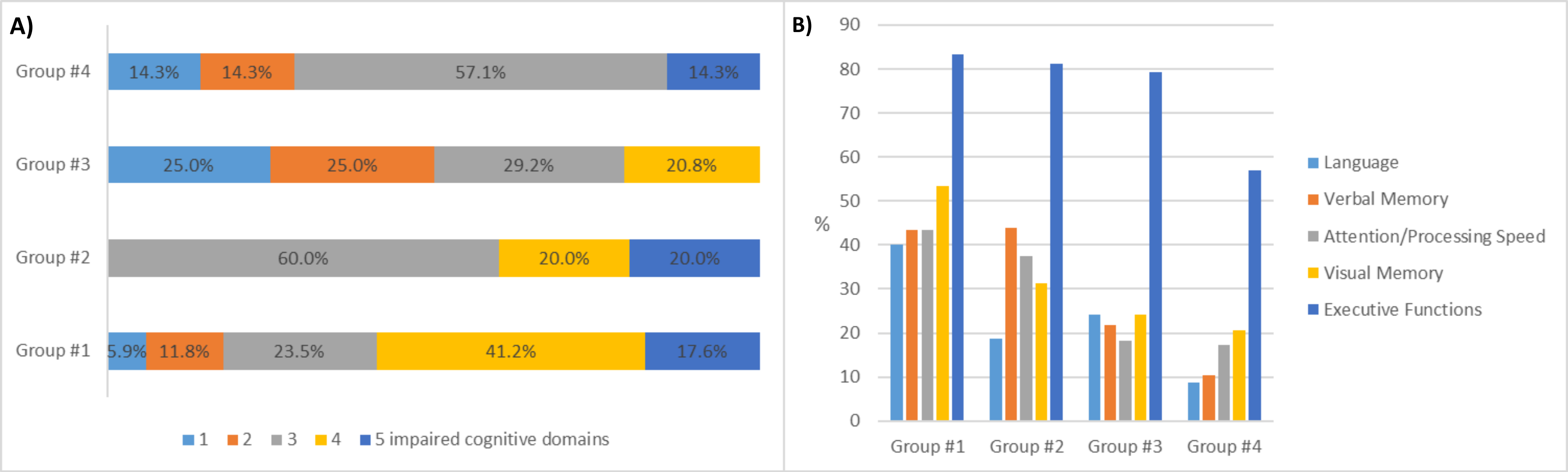

Figure S6. Impaired cognitive domains in each group of participants: **A)** Percentages of participants presenting 1, 2, 3, 4, and 5 impaired cognitive domains in each group; **B)** percentages of participants in each group presenting impairment in the cognitive domains assessed with the neuropsychological battery of tests.
